# Structure of a Spumaretrovirus Gag Central Domain Reveals an Ancient Retroviral Capsid

**DOI:** 10.1371/journal.ppat.1005981

**Published:** 2016-11-09

**Authors:** Neil J. Ball, Giuseppe Nicastro, Moumita Dutta, Dominic J. Pollard, David C. Goldstone, Marta Sanz-Ramos, Andres Ramos, Erik Müllers, Kristin Stirnnagel, Nicole Stanke, Dirk Lindemann, Jonathan P. Stoye, William R. Taylor, Peter B. Rosenthal, Ian A. Taylor

**Affiliations:** 1 Macromolecular Structure Laboratory, The Francis Crick Institute, Mill Hill Laboratory, London, United Kingdom; 2 Structural Biology of Cells and Viruses, The Francis Crick Institute, Mill Hill Laboratory, London, United Kingdom; 3 Retrovirus-Host Interactions Laboratory, The Francis Crick Institute, Mill Hill Laboratory, London, United Kingdom; 4 Institute of Virology, Technische Universität Dresden, Dresden, DE; 5 Faculty of Medicine, Imperial College London, London, United Kingdom; 6 Computational Cell and Molecular Biology Laboratory, The Francis Crick Institute, Mill Hill Laboratory, London, United Kingdom; Universitätklinikum Heidelberg, GERMANY

## Abstract

The *Spumaretrovirinae*, or foamy viruses (FVs) are complex retroviruses that infect many species of monkey and ape. Despite little sequence homology, FV and orthoretroviral Gag proteins perform equivalent functions, including genome packaging, virion assembly, trafficking and membrane targeting. However, there is a paucity of structural information for FVs and it is unclear how disparate FV and orthoretroviral Gag molecules share the same function. To probe the functional overlap of FV and orthoretroviral Gag we have determined the structure of a central region of Gag from the Prototype FV (PFV). The structure comprises two all α-helical domains NtD_CEN_ and CtD_CEN_ that although they have no sequence similarity, we show they share the same core fold as the N- (NtD_CA_) and C-terminal domains (CtD_CA_) of archetypal orthoretroviral capsid protein (CA). Moreover, structural comparisons with orthoretroviral CA align PFV NtD_CEN_ and CtD_CEN_ with NtD_CA_ and CtD_CA_ respectively. Further *in vitro* and functional virological assays reveal that residues making inter-domain NtD_CEN_—CtD_CEN_ interactions are required for PFV capsid assembly and that intact capsid is required for PFV reverse transcription. These data provide the first information that relates the Gag proteins of *Spuma* and *Orthoretrovirinae* and suggests a common ancestor for both lineages containing an ancient CA fold.

## Author Summary

Foamyviruses (FVs) or Spuma-retroviruses derive their name from the cytopathic effects they cause in cell culture. However, infection in humans is benign and FVs have entered the human population through zoonosis from apes resulting in the emergence of Prototype FV (PFV). Like all retroviruses, FVs contain *gag*, *pol* and *env* structural genes and replicate through reverse-transcription and host genome integration. Gag, the major structural protein, is required for genome packaging, virion assembly, trafficking and egress. However, although functionally equivalent, FV and orthoretroviral Gag share little sequence homology and it is unclear how they perform the same function. Therefore, to understand more about relationship between FV and orthoretroviral replication we have carried out structural studies of PFV-Gag. Here we present the structure of CA domains from a central region PFV-Gag and show that despite little sequence similarity they share the same fold as the CA domains of orthoretroviral Gag. These data provide the first information relating the *Spuma* and *Orthoretrovirinae* Gag proteins. We discuss our findings in terms of evolutionary divergence of spuma and orthoretroviral lineages.

## Introduction

Spuma- or foamy viruses (FVs) are complex retroviruses that constitute the only members of the *Spumaretrovirinae* subfamily within the *Retroviridae* family [1]. They have been isolated from a variety of primate hosts [2–5] as well as from cats [6–8], cattle [9], horses [10] and sheep [11]. Endogenous FVs have also been described in sloth [12], aye-aye [13] and coelacanth [14]. Prototype foamyvirus (PFV) is a FV isolated from human sources [15, 16]. The PFV genome is highly similar to that of simian foamy virus isolates from chimpanzee (SFV_cpz_) and so infection in humans is believed to have arisen through zoonotic transmission [17–19]. Nevertheless, even though FVs are endemic within non-human primates and display a broad host range, human-to-human transmission of PFV has never been detected. Moreover, although in cell culture FV infection causes pronounced cytopathic effects [20], infection in humans and natural hosts is apparently asymptomatic [21–23] making their usage as vectors for gene therapy an attractive proposition [24].

FVs share many similarities with other retroviruses in respect of their genome organisation and life cycle. However, they vary from the *Orthoretrovirinae* in a number of important ways. These include the timing of reverse transcription that occurs in virus producer cells rather than newly infected cells [25, 26] and the absence of a Gag-Pol fusion protein [27, 28]. In addition, the Gag protein remains largely unprocessed in FVs [29] whereas within the *Orthoretrovirinae* processing of the Gag polyprotein represents a critical step in viral maturation, producing the internal structural proteins Matrix (MA), Capsid (CA) and Nucleocapsid (NC) found in mature virions. Furthermore, FV Gag lacks the Major Homology Region (MHR) and Cys-His boxes found in orthoretroviral CA and NC, respectively. Despite these profound dissimilarities, the Gag proteins of the two retroviral subfamilies carries out the same functional roles including viral assembly, nucleic acid packaging, transport to and budding through the cytoplasmic membrane of the producer cell as well as trafficking through the cytoplasm of the target cell and uncoating. In addition, FV Gag also contains the determinants for restriction by Trim5α [30, 31] that in orthoretroviruses comprises the assembled CA lattice [32].

To date, high-resolution X-ray and/or NMR structures have been reported for MA, CA and NC components of Gag from numerous retroviruses [33–42] but among FVs only the structure of the Env-binding N-terminal domain of PFV-Gag has been reported [43]. Further structural information with regard to other Gag domains of FVs has remained elusive but is vital for any detailed understanding of how FV Gag fulfils its many functions. Here we report the structure and present structure/function studies of a di-domain from the central region of PFV-Gag. Our data reveal that although unrelated at the level of primary sequence, FV central domains are structurally related to the N- and C-terminal domains of orthoretroviral CA. Moreover, they share the capacity for self-association and are required for virion capsid assembly and viral infectivity. Further phylogenetic and combined comparative structural analysis reveals FV central domains also have the same organisational arrangement as orthoretroviral CA and we propose that both arose through genetic divergence from a common, double domain ancestor.

## Results

### Structure of the PFV-Gag central conserved region

**Fig 1:**
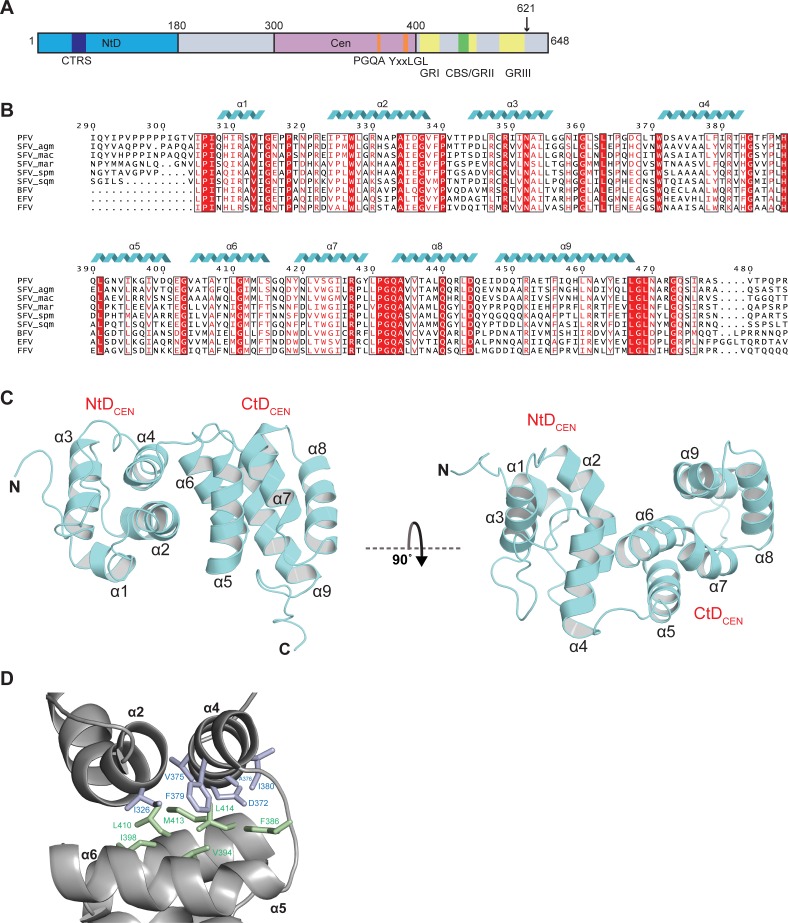
NMR structure of the central domain of PFV Gag. (**A**) Schematic representation of PFV Gag. Regions corresponding to the Gag-NtD and Gag-central domains are coloured cyan and magenta respectively. Sequence motifs and conserved regions are highlighted cytoplasmic targeting and retention sequence (CTRS) (blue), PGQA and YxxLGL (orange), Chromatin binding sequence (CBS) (green) and GR boxes (yellow). The Gag processing cleavage site is indicated with an arrow. (**B**) Sequence alignment of foamy virus Gag-central domains from mammals, old and new world monkeys (SFV). Mammalian FVs are abbreviated as follows: BFV, Bovine; EFV, Equine; FFV, Feline. Monkey species are abbreviated as follows: mac, Macaque; agm, African green monkey; spm, Spider monkey; sqm, Squirrel monkey; mar, marmoset. Numbering corresponds to the PFV sequence. Cartoons (cyan coils) above the alignment indicate the position of α-helices in the PFV-Gag NtD_CEN_ and CtD_CEN_ domain structures. The regions with greatest sequence homology are boxed and highlighted and residues that are conserved in all sequences are also coloured white. (**C**) Cartoon representation of PFV-Gag(300–477) backbone is shown in cyan. The secondary structure elements are numbered sequentially from the amino-terminus and the N- and C-termini are indicated. Helices α1 to α4 and α5 to α9 that comprise NtD_CEN_ and CtD_CEN_ respectively are indicated in the left and right hand panels. (**D**) The PFV-Gag NtD_CEN_ and CtD_CEN_ interface. The protein backbone is shown in grey cartoon representation. NtD_CEN_ and CtD_CEN_ α-helices that pack at the interface are labelled. Residues that make hydrophobic contacts are shown as sticks, blue from NtD_CEN_ and green from CtD_CEN_.

Alignment of the primary sequences of FV Gag proteins from primate and other mammalian hosts reveals two regions of strong conservation, an N-terminal region corresponding to the Env-binding domain [43–45] containing the cytoplasmic targeting and retention sequence (CTRS) [46, 47] and the other located centrally containing highly conserved PGQA and YxxLGL sequences [48] and just N-terminal to the chromatin binding sequence (CBS) [49]and GR boxes [50] (**Fig 1A**). Within this central region, large sections of highly conserved sequence are present (**Fig 1B**). Therefore, to understand more about the nature of the PFV-Gag central conserved region, the structure of PFV-Gag(300–477) was determined in solution using multidimensional heteronuclear NMR spectroscopy. Details of data collection, structure determination and model quality are presented in **Table 1.**

**Table 1:** NMR and refinement statistics for PFV-Gag Central domains.

	PFV-Gag (300–477)	PFV-Gag CtD_CEN_
**NMR distance and dihedral constraints**		
NOE Distance constraints		
*Total NOE*	4140	3061
*Unambiguous*	3637	2616
*Intermolecular*		20x2
Hydrogen bonds	48	31x2
Total dihedral angle restraints		
*Φ*	113	74x2
*Ψ*	114	74x2
Total RDCs	46	41x2
**Structure statistics**		
Violations (mean and s.d.)		
*Distance constraints (>0*.*5Å)*	0	0
Deviations from idealised geometry		
*Bond lengths (Å)*	0.015	0.008
Average pairwise r.m.s. deviation (Å)		
*Heavy*	0.6	0.6

The structure comprises two all helical domains, connected by a short 5-residue linker (**Fig 1C**). Residues P300-H383 make up the N-terminal domain (PFV-NtD_CEN_) containing four helices (α1-α4) and the C-terminal domain (PFV-CtD_CEN_), residues H389-R477, contains the remaining five helices (α5-α9). Superposition of the 20 conformers in the family of structures results in a backbone atom rmsd of 0.3 Å for ordered residues 304–355, 358–477 showing that the structure is well defined except for the N- and C-termini and loop regions (**S1A Fig**). In PFV-NtD_CEN_, helices α1-α3 form an antiparallel 3-helix bundle connected to α4 by a long loop that closely tracks one face of α3. In PFV-CtD_CEN_ helices α5-α9 are arranged as a five-helix antiparallel bundle. In both domains, the inner faces of the helices pack to form an extensive hydrophobic core through interaction of apolar sidechains.

Examination of the protein backbone dynamics using ^15^N NMR relaxation measurements (**S1B Fig**), show that residues within helices α1- α4 and α5-α9 of the PFV-NtD_CEN_ and PFV-CtD_CEN_ exhibit large and positive heteronuclear NOE (HetNOE) values and have uniform ^15^N-T_1_ and -T_2_ values indicating a rigid backbone. Additionally, the presence of inter-domain NOEs, together with little variation in the T_1_/T_2_ values, suggests the PFV-NtD_CEN_ and PFV-CtD_CEN_ are structurally and dynamically dependent and have a coupled movement. Based on these relaxation rates and assuming an isotropic model, a rotational correlation time (t_c_) of 14.1 ns for the NtD_CEN_-CtD_CEN_ di-domain was determined, consistent with a ~ 20 kD globular protein. The residues at the N- and C-termini outside of this core region have lower T_1_ and higher T_2_ values, reduced or negative HetNOEs, close to zero ^1^D_NH_ residual dipolar couplings (RDC) and mainly random coil chemical shifts indicating rapid (psec) internal motion in these terminal regions. In addition, the relaxation data also reveals internal regions of high mobility, including residues G356 to G366 located in the long loop connecting α3-α4, residues G384 to P388 in the NtD_CEN_-CtD_CEN_ interdomain linker and G432 part of a stretch of highly conserved residues (-P_431_-**G**-Q-A_434_-) located in the loop connecting α7-α8 of CtD_CEN_ and in close spatial proximity to the conserved Y/F_464_-x-x-L-G-L_469_ motif (**Fig 1A and 1B**), at the C-terminus of α9 that is required for Gag assembly [48]. Together with these relaxation data a number of interdomain NOEs (**S1C Fig**) define a largely hydrophobic NtD_CEN_-CtD_CEN_ interface comprising 550Å^2^ of buried surface area (**Fig 1D**). Although not extensive in area, there is substantial packing of apolar sidechains from NtD_CEN_ residues on helices α2 and α4 (I326, V375 and F379) with CtD_CEN_ residues (V394, I398, L410, M413 and L414) on helices α5 and α6 (**Fig 1D**) that contribute to the stability of the interface.

### Structural similarity with CA of other retroviral genera

**Fig 2:**
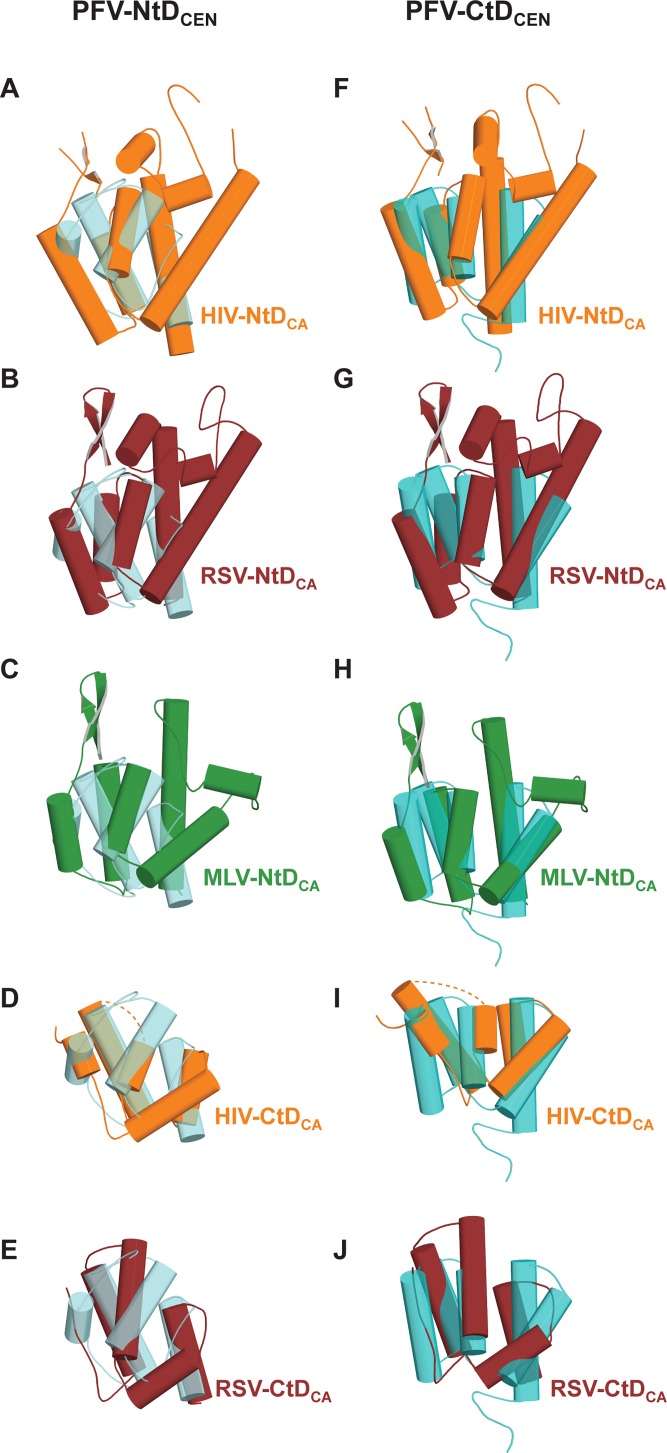
Alignment of PFV_CEN_ domains with orthoretroviral CA proteins. Panels **A-E**, are best-fit 3D structural superimpositions of PFV-NtD_CEN_ (light cyan) with (**A**) HIV-1 NtD_CA_ (orange), (**B**) RSV NtD_CA_ (maroon), (**C**) MLV NtD_CA_ (green), (**D**) HIV-1 CtD_CA_ (orange) and (**E**) RSV CtD_CA_ (maroon). Panels **F-J** are best-fit 3D structural superimpositions of PFV-CtD_CEN_ (dark cyan) with (**F**) HIV-1 NtD_CA_ (orange), (**G**) RSV NtD_CA_ (maroon), (**H**) MLV NtD_CA_ (green), (**I**) HIV-1 CtD_CA_ (orange) and (**J**) RSV CtD_CA_ (maroon). In all panels, molecules are shown in cartoon representation with α-helices displayed as cylinders. The dashed lines in panels D and I indicate the connectivity between helices 8 and 9 of HIV-CTD_CA_ that is disordered in the CA hexamer structures used in the alignment.

Initial structural similarity searches of the PDB with PFV-Gag(300–477), PFV-NtD_CEN_ and PFV-CtD_CEN_ were conducted using the SSM server [51]. Application of this approach, produced only very weak matches based on the quality of alignment Q-scores (0.1–0.3). Nevertheless, 11 of the top 15 alignments for individual NtD_CEN_ and CtD_CEN_ domains were with either amino- (NtD_CA_) or carboxyl-terminal domains (CtD_CA_) from orthoretroviral CAs (**S1 Table**). However, although matches were found for NtD_CEN_ with orthoretroviral NtD_CA_ domains and for CtD_CEN_ with orthoretroviral CtD_CA_ domains and the helical connectivity and topological arrangement of secondary structures were largely conserved (**S2 Fig**), notably some top alignments were between NtD_CEN_ and CtD_CA_ domains and by CtD_CEN_ with NtD_CA_ domains, **Fig 2.**

Inspection of these alignments reveals a closest match for PFV-Gag NtD_CEN_ with the CtD_CA_ of the alpha-retrovirus RSV (3G1G) based upon rmsd over all aligned α-carbons. However, in all these alignments the orthoretroviral CtD_CA_ structures contain an additional α-helix that inserts between α3 and α4 of NtD_CEN_ (**Fig 2D and 2E**). Structural alignments with orthoretroviral NtD_CA_, reveal the closest match is between PFV-Gag CtD_CEN_ and the NtD_CA_ of the gamma-retrovirus MLV (3BP9) (**Fig 2H**). Again, however, although the core fold aligns well, the interspersing loops that connect the secondary structure elements in the orthoretroviral NtD_CA_ are absent or much shorter in PFV-Gag CtD_CEN_.

These data provide evidence for a structural conservation between orthoretroviral CA and spumaretroviral Gag but these very weak alignments do not discriminate well between NtD_CEN_−NtD_CA_, CtD_CEN_−CtD_CA_ (forward; NN, CC) and NtD_CEN_−CtD_CA_, CtD_CEN_−NtD_CA_ (reverse; NC, CN) pairings. Therefore, to assess the significance and quantify the degree of similarity for forward and reverse pairings we applied a structural alignment method based on the generation of a population of 'decoy' models to provide a background distribution of scores [52] combined with structural superposition using the SAP program [53]. This method has the advantage that it uses a local structural environment-based alignment and that each comparison in the random pool is between two models of the same size and secondary structure composition as the pair of native structures being investigated.

For this analysis five orthoretroviral CA proteins were chosen where both NtD_CA_ and CtD_CA_ structures were available. Individual CA domains were then compared with both PFV-Gag NtD_CEN_ and CtD_CEN_ and the associated decoy models. The degree of similarity between the domains with respect to the bulk alignments with decoy models ranged from < 2σ to > 5σ (Z-score). However, as with the SSM searches significant 4σ results were obtained for both reverse as well as forward alignments, **Table 2**. Of the top five Z-scores in **Table 2**, four are associated with N-N and C-C pairings. Although this does suggest conventional forward linear domain equivalence, in order to obtain a more quantitative consensus for forward versus the reverse domain pairings, the Z-scores for each domain pairing were combined using a T-test statistic over all five viruses. Employing this analysis, all four possible domain pairings were significant with probabilities (T_prob_) ranging from 10^−6^ to > 10^−18^. However, the two reversed pairings (NC and CN) have lower probabilities than the forward pairings (NN and CC) **Table 2** and by combining the probabilities log_10_(T_prob_NN.T_prob_CC)–log_10_(T_prob_NC.T_prob_CN) a 12-log difference-probability (ΔT_prob_) is now apparent for the forward pairing with respect to the reverse.

**Table 2:** Z-score and T-test significance of SAP alignments.

	Ortho N_CA_		Ortho C_CA_	
Virus	Spuma-N	Spuma-C	Spuma-N	Spuma-C
BLV	^1^ **4.49**	3.67	3.40	**4.05**
HIV-1	3.70	3.69	3.76	3.36
HML2	2.17	**4.59**	3.02	3.90
HTLV-1	**4.03**	**4.01**	3.85	2.81
RSV	3.12	3.54	3.75	**5.01**
^2^T_prob_	9.47e^-15^	1.49e^-6^	5.31e^-15^	1.32e^-18^
-log_10_(T_prob_)	15	6	15	18
^3^-log_10_(ΔT_prob_)	12	/	/	/

^1^Z score. Pairings with Z > 4 are in highlighted in bold

^2^Student’s T-test probability

^3^Differential probability = log_10_(T_prob_NN.T_prob_CC)–log_10_(T_prob_NC.T_prob_CN)

Both the T and Z statistics support an ancestral relationship between the central domains of PFV-Gag and the NtD_CA_ and CtD_CA_ of orthoretroviral CA. This suggested forward pairing (NN and CC) would support the notion that the orthoretroviral CA and PFV-Gag NtD_CEN_-CtD_CEN_ arose through genetic divergence from a common, double domain ancestor without a requirement for transposition.

### Oligomerisation state of foamy virus Gag-central domains

Given the requirement for CA oligomerisation in orthoretroviral Gag assembly and maturation, the self-association and assembly properties of PFV-Gag(300–477), PFV-NtD_CEN_ and PFV-CtD_CEN_ were analysed by sedimentation velocity (SV) and equilibrium (SE) analytical ultracentrifugation (AUC). The experimental parameters, molecular weights derived from the data and statistics relating to the quality of fits are shown in **Table 3**.

**Table 3:** Hydrodynamic parameters of PFV-Gag Central domains.

	PFV-Gag	PFV-Gag	PFV-Gag	PFV-Gag
	(300–477)	NtD_CEN_	CTD_CEN_^mon^	CTD_CEN_^dim^
***Parameter***				
$\bar{v}$ (ml.g^-1^)	0.7416	0.7415	0.7312	
ρ(g.ml^-1^)	1.005	1.005	1.005	
^a^ M_r_ (Da)	20,543	10,660	11,894	
ε_280_ (M^-1^.cm^-1^)	16,960	11,000	5,960	
***Sedimentation velocity***				
C_range_ (μM)	24–97	188	42–168	
^b^ S_20,w_ (x10^13^) sec	1.87	1.25	1.65	2.07
^c^M_w_ C(S) (kDa)	20.6	10.3	14.7	20.7
^d^ rmsd C(S) (x10^-3^)	3.0–5.1	5.7	5.8–7.6	
***Sedimentation equilibrium***				
C_range_ (μM)	24–97		17–84	
^e^K_D_ (μM)	-		0.9	
^f^ M_w_ (kDa)	20.3		-	
^g^ rmsd (x10^-3^)	6.9–7.1		4.8–6.0	
^h^ χ^2^	1.98		1.33	

^a^Molar mass calculated from the protein sequence

^b^The S_20,w_ value remained constant across the concentration range tested.

^c^The weight averaged molecular weight derived from the best fit C(S) function.

^d^The range of the rms deviations observed when data were fitted using a continuous sedimentation coefficient distribution model.

^e^The equilibrium dissociation constant calculated from a monomer-dimer self-association model.

^f^The weight averaged molecular weight from Global SE analysis using a species analysis model.

^g^The range of the rms deviations observed for each multi-speed sample when fitted individually to the appropriate model.

^h^The global reduced chi-squared for the global fit to the appropriate model.

**Fig 3:**
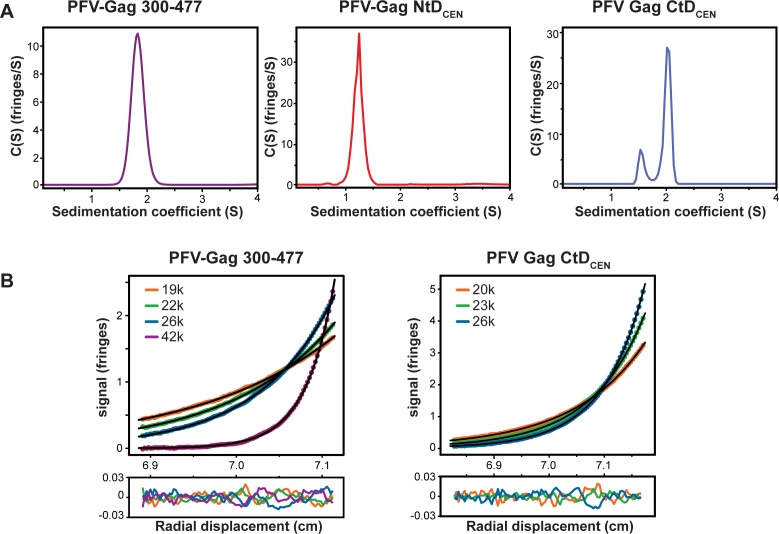
Conformation and solution oligomeric state of FV-Gag central domains. (**A**) C(S) distributions derived from sedimentation velocity data recorded from PFV-Gag(300–477) at 1 mg/mL (left panel); PFV-Gag NtD_CEN_ at 2 mg/mL (middle panel) and PFV-CtD_CEN_ 2 mg/mL (right panel). (**B**) Multi-speed sedimentation equilibrium profiles determined from interference data collected on PFV-Gag(300–477) at 49 μM (left panel) and PFV-Gag CtD_CEN_ at 42 μM (right panel). Data was recorded at the speeds indicated. The solid lines represent the global best fit to the data using either a single species or monomer-dimer equilibrium model. The lower panels show the residuals to the fit.

SV-AUC analysis of the whole of the conserved region, PFV-Gag(300–477), revealed a sedimentation coefficient (S_20,w_) of 1.87 (**Fig 3A**) and derived molar mass of 20.6 kDa demonstrating that PFV-Gag(300–477) is a stable monomer in solution. These observations were confirmed by multispeed SE-AUC at varying protein concentration. The equilibrium distribution from an individual multispeed experiment is presented in **Fig 3B**. The individual gradient profiles showed no concentration dependency of the molecular weight and fit globally with a single ideal molecular species model, producing weight averaged molecular weight of 20.3 kDa demonstrating the monomeric nature of this PFV central region. SV-AUC analysis of PFV-Gag NtD_CEN_ measured at high protein concentration (188 μM) also revealed this domain to be monomeric in solution with a only a single species, (S_20,w_) of 1.25 (**Fig 3A**) with derived molar mass of 10.3 kDa present (**Table 3**). By contrast SV-AUC data recorded on PFV-Gag CtD_CEN_ produced a sedimentation coefficient continuous distribution function, C(S), that contained two species with S_20,w_ of 1.65 and 2.07 with derived molecular weights of 14.7 kD and 20.7 kD (**Table 3 and Fig 3A**). Notably, the proportion of the fast 2.07 S, component increased with increasing concentration (**S3 Fig**) consistent with monomer-dimer equilibrium. Therefore, in order to quantify the affinity and stoichiometry of self-association, multispeed SE-AUC recorded at varying protein concentration was employed. These data (**Fig 3B**) are best fit by a monomer-dimer self-association model where the 11.9 kDa PFV-Gag CtD_CEN_ monomers dimerise with an equilibrium association constant of 1.1x10^6^ M^-1^ (0.9 μM K_D_). These data are consistent with the distribution of peaks in the C(S) functions derived from SV-AUC data. Moreover, they reveal that whilst the entire PFV-Gag central region is monomeric PFV-Gag CtD_CEN_ has the propensity for self-association.

### The PFV-Gag CtD_CEN_ homodimer

**Fig 4:**
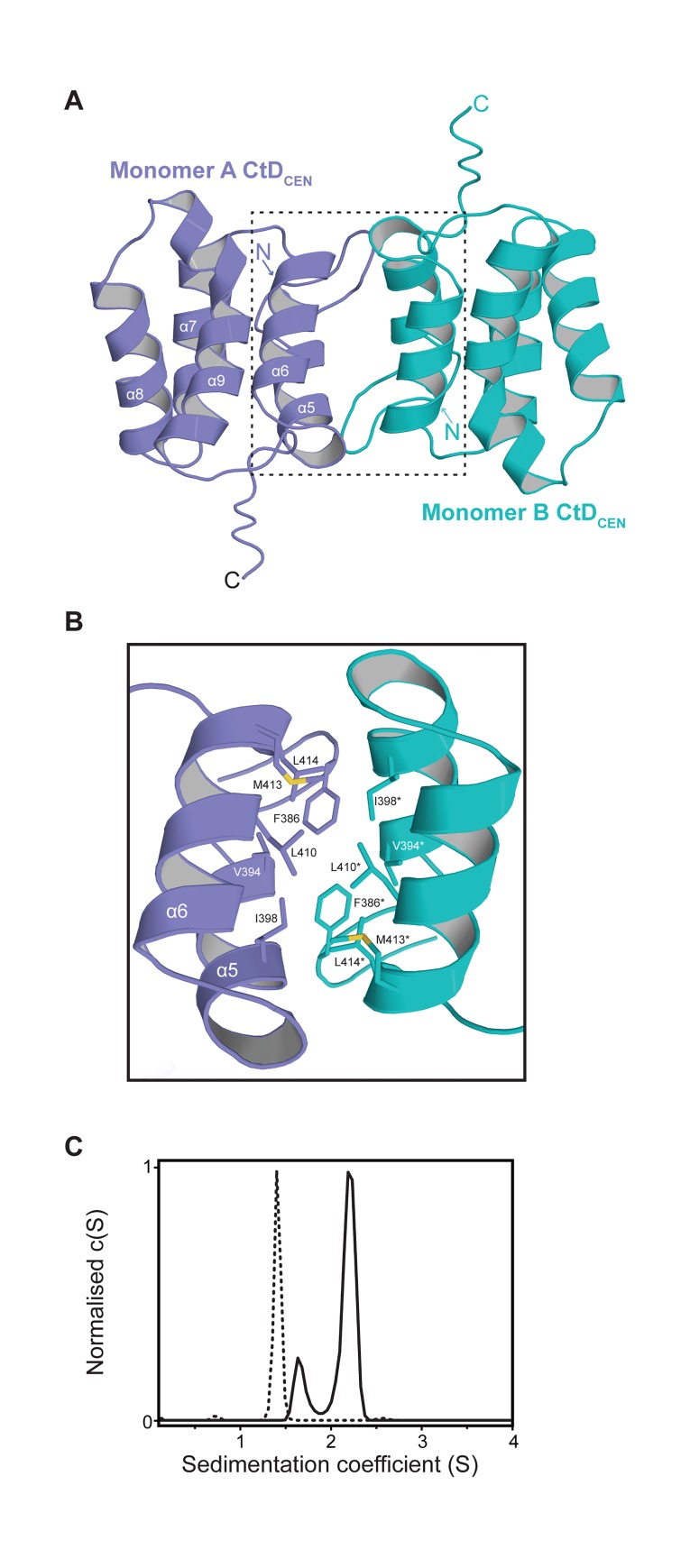
NMR structure of PFV-Gag CtD_CEN_ homodimer. (**A**) Cartoon representation of the structure of the PFV-Gag CtD_CEN_ dimer. Monomer-A is shown in dark blue and Monomer-B in cyan. The α-helices are labelled as for PFV-Gag(300–477) and the N- and C-termini of each monomer are indicated. (**B**) Details of the homodimer interface. Residues that contribute to the interface are shown as sticks. (**C**) C(S) distribution derived from sedimentation velocity data recorded from PFV-Gag CtD_CEN_ L410E/M413E mutant at 2 mg/mL (dashed line). The C(S) distribution derived from sedimentation velocity data recorded from *wt* PFV-Gag CtD_CEN_ at 2 mg /mL is shown also for comparison (solid line).

Given the dimerisation properties of PFV-Gag CtD_CEN_ and the structural homology with self-associating orthoretroviral CA-domains we determined the solution structure of the PFV-Gag CtD_CEN_ homodimer. Details of data collection and structure determination are presented in **Table 1**. Superposition of the 20 conformers in the family of structures (**S4A Fig**) results in a backbone atom rmsd of 0.3 Å for ordered residues 381–477 revealing a well-defined structure except for residues close to the N- and C-termini. In the structure, **Fig 4A**, each monomer comprises five-antiparallel α-helices (residues N393-E402, V404-L414, Q420-Y429, Q433-Q445 and Q450-L467) and is virtually identical to the equivalent helices, α5 to α9, in PFV-Gag(300–477) with the exception that α5 is ~2 turns shorter. Analysis of NMR relaxation data (**S4B Fig**) reveals little variation in T_1_/T_2_ values and the derived rotational correlation time (t_c_) of 18.2 ns is consistent with a ~ 24 kD CtD_CEN_ homodimer.

The homodimer interaction is defined by numerous NOEs (**S4C Fig**) and encompasses 470 Å^2^ of buried surface. The interface is largely hydrophobic with the majority of interactions resulting from packing of α6 of one monomer against α6 of the opposing monomer together with some contribution from hydrophobic side chains of residues on α5 (**Fig 4B**). At the centre of the interface the side chains of I398, L410 and M413 from one monomer pack against I398*, L410* and M413* of the opposing monomer and comprise a continuous apolar network. Disruption of this network by introduction of an L410E/M413E double mutation results in total loss of dimerisation as revealed by SV-AUC analysis (**Fig 4C).** Notably, I398, L410 and M413 are also involved in the NtD_CEN_-CtD_CEN_ interface were they make apolar contacts with side chains of residues on α2 and α4 in NtD_CEN_ (**Fig 1D)**.

### NtD_CEN_-CtD_CEN_ interface mutations effect virus infectivity and particle morphology

**Fig 5:**
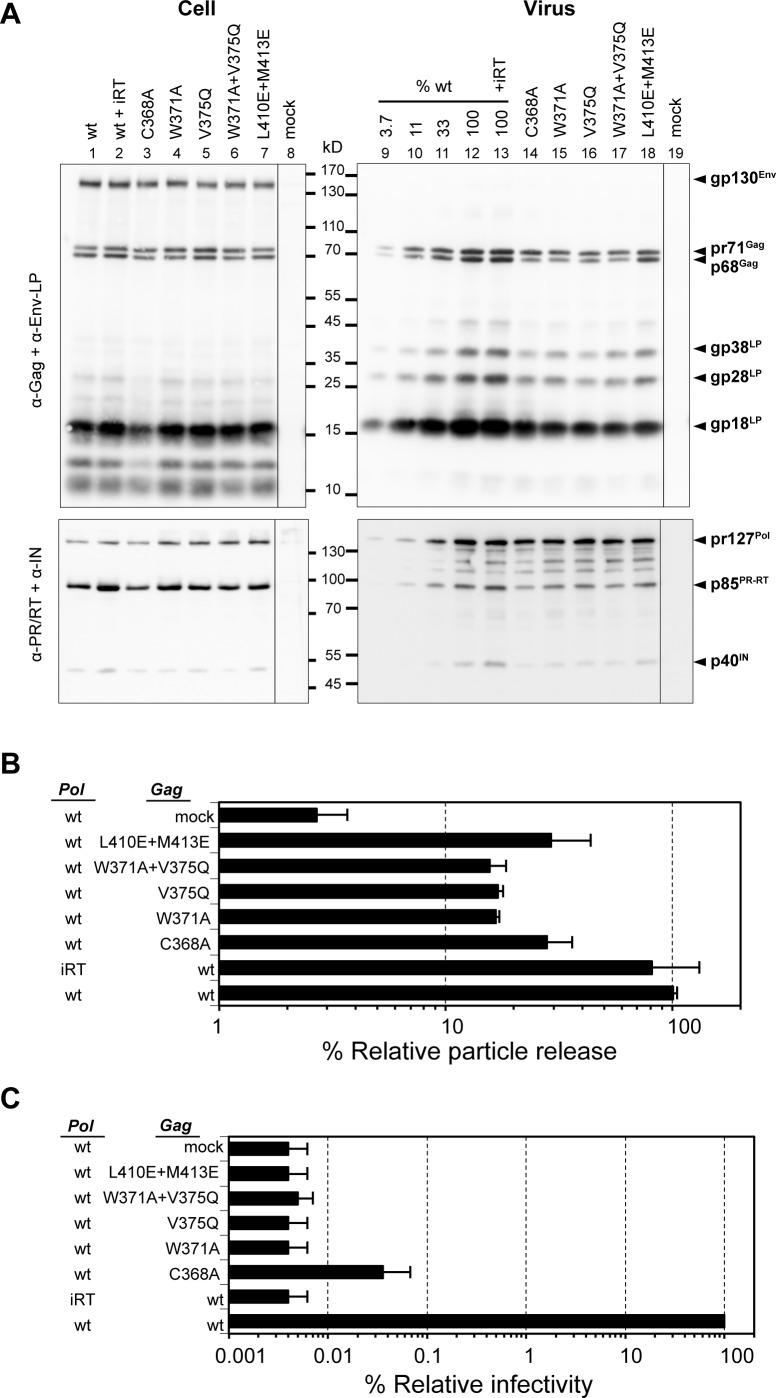
Particle production and infectivity of PFV-Gag central domain mutants. (**A**) Western blot analysis of producer cell lysates (**Cell**) and pelleted viral supernatants (**Virus**) with polyclonal antibodies specific for PFV-Gag (α-Gag) and PFV Env-LP (α-Env-LP) or monoclonal antibodies specific for PFV-PR/RT (α-PR/RT) and integrase (α-IN). Residue substitutions in Gag are indicated above each track, (*wt*) wild type virus, (*wt +iRT*) wild type virus with defective reverse transcriptase. In the right-hand panel %*wt* are different *wt* control loadings and arrows indicate the migration of Gag, Env and Pol proteins. (**B**) Relative amounts of released Gag quantified from Western blots data from two independent experiments. (**C**) Relative infectivity of extracellular 293T cell culture supernatants using an eGFP marker gene transfer assay, determined 3 days post infection. Means and standard deviations of three independent experiments are shown. The values obtained using the wild type Gag packaging vector were arbitrarily set to 100%. Absolute titres of these supernatants were 1.8 x 10^6^ to 1.1 x 10^7^ ffu/ml.

To probe the function of domain interface residues in a virological context, V375Q and L410E/M413E amino acid interface-disrupting mutations were introduced into PFV-Gag in a mammalian virus expression system. In addition, W371A or C368A alanine substitution mutations designed to disrupt hydrophobic packing of the Gag-NtD_CEN_ domain were also made along with particles lacking reverse transcriptase (iRT). The effects of these substitutions on virus Gag/Env/Pol processing, particle production, and infectivity were then assessed (**Fig 5)**. In all instances, viral particles were produced and the composition and processing of Gag Pol and Env was comparable with *wt* PFV (**Fig 5A)**, although, overall particle production was reduced between 3–5 fold, in all of the mutants (**Fig 5B).** In contrast to these small particle production defects, viral infectivity upon introduction of V375Q and L410E/M413E interface mutations was reduced by over 4 orders of magnitude (**Fig 5C)** comparable with 3–4 log reductions observed in W371A and C368A NtD_CEN_ disruption mutants and 4 log reductions observed with a combined W371A/V375Q mutant or a Gag *wt* /Pol iRT virus.

**Fig 6:**
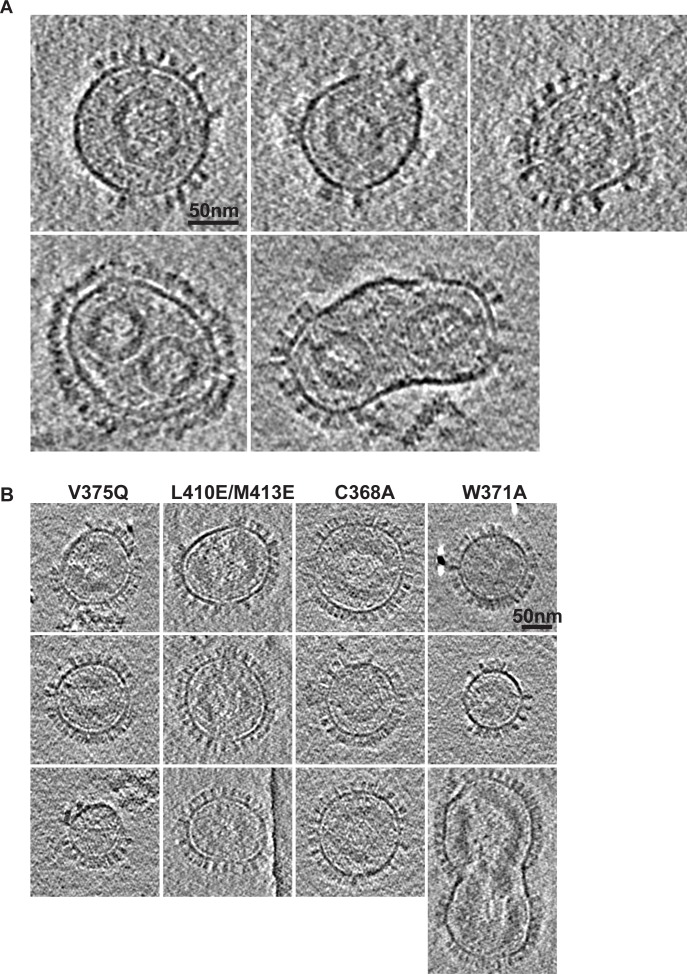
Cryo-electron microscopy analysis of wt and mutant PFV particles. (**A**) 5 nm thick slices of electron cryotomograms of five individual *wt* PFV particles. (**B**) 5 nm thick slices of electron cryotomograms of four PFV-Gag central domain mutants. Three representative images of each mutant are shown. Scale bars are 50 nm.

Given these large effects on viral infectivity, the morphology and integrity of particles was also assessed by cryo-electron microscopy (cEM) (**Fig 6)**. Analysis of *wt* PFV (**Fig 6A and S2 Table**) reveals roughly spherical 1000 to 1300 Å diameter particles with external spikes of the Env protein and core structures as previously described [45, 54]. We performed cryo-tomography to study virus particles in 3-dimensions. The majority of particles contain a dense core structure, 600 to 800 Å, in their interior. In some instances, two cores were present, often correlating with a larger virion size, as observed with other foamy virus [45] and orthoretroviral particles [55]. Inspection of the core morphology revealed that it comprised an 80–100 Å layer that is strongly faceted and contains vertices indicative of a polyhedral structure with underlying icosahedral order. By contrast, although of similar size and displaying Env spikes, no virus particles with V375Q and L410E/M413E interface mutations contained an internal dense core, indicating they have defects in core assembly (**Fig 6B and S2 Table**). The particles appear either empty or in some cases contain a diffuse layer of density close to the inner side of viral envelope. Similarly, particles of NtD_CEN_ disruption mutants C368A and W371A also have *wt* size distribution and external morphology but have no cores (**Fig 6B and S2 Table**) demonstrating that mutations affecting NtD_CEN_—CtD_CEN_ interactions and those designed to interfere with Gag central domain folding are both deleterious to core assembly.

**Fig 7:**
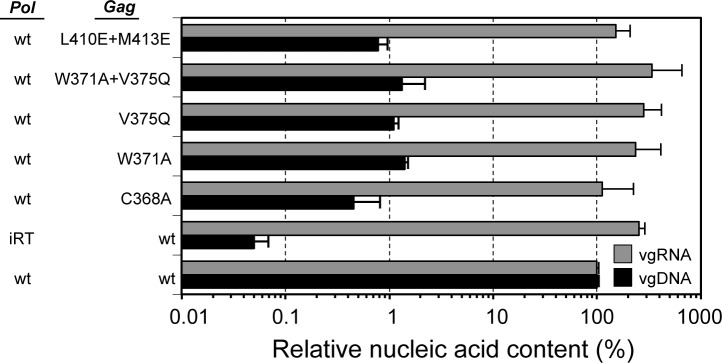
Analysis of genome content of PFV-Gag central domain mutants. Quantification of particle-associated PFV genomic RNA (vgRNA) and DNA (vgDNA) by qPCR. Mean values and standard deviation (n = 2) normalized for Gag content are shown as relative values compared to the *wt* control.

The effects of the interface and NtD_CEN_ disruption mutations on reverse-transcription of the viral genome were also examined by qPCR. These data (**Fig 7**) revealed that all particles contained similar levels of PFV RNA suggesting that there was no requirement for an assembled viral core to recruit and/or package RNA genomes. However, quantitation of viral DNA revealed that in both the interface or Gag-NtD_CEN_ disruption mutants that lack cores, there was a 100-fold reduction in the DNA genome content. The DNA genome content of the iRT mutant was reduced 1000-fold. Given that reverse transcriptase is recruited into particles in the mutants with a comparable efficiency to *wt* (**Fig 5A**) these data reveal a requirement for core formation in order for efficient reverse transcription to occur.

## Discussion

### The foamy virus Gag central domain is related to orthoretroviral CA

Gag is the major structural protein of both spuma and orthoretroviral subfamilies, required for viral assembly, genome packaging and budding from producer cells [56]. Nevertheless, despite the conservation of function, spuma and orthoretroviral Gag share little if any sequence identity [57]. Any relatedness in terms of structure therefore remains unclear. Previous studies have shown that an N-terminal domain from spumaretroviral Gag (PFV-Gag-NtD), whilst possessing some of the functional properties of orthoretroviral Gag MA and CA maturation products, is entirely unrelated on a structural level [43]. We have now determined the solution structure of a central region of PFV-Gag (NtD_CEN_-CtD_CEN_). By contrast with the N terminal region, this structure reveals that the central region of spumaretroviral Gag has unanticipated structural similarity to the NtD_CA_ and CtD_CA_ of orthoretroviruses. The NtD_CEN_ and CtD_CEN_ domains comprise 4 and 5 helical bundles, respectively, that in terms of topology align well with secondary structure elements of NtD_CA_ and CtD_CA_ domains. However, overall the alignment is relatively weak and although the core helical bundles are structurally very similar, the orthoretroviral NtD_CA_ and CtD_CA_ contain additional helices and loop insertions. We therefore applied an unbiased objective approach to assess the degree of similarity between PFV-NtD_CEN_ and PFV-CtD_CEN_ with NtD_CA_ and CtD_CA_ domains [52, 53]. This analysis confirmed the relationship between the spuma- and orthoretroviral sequences and revealed that by far the preferred statistical alignment was also the most plausible on biological grounds, specifically a “forward pairing” where PFV-NtD_CEN_ corresponds to NtD_CA_ and PFV-CtD_CEN_ relates to CtD_CA_. Based on these observations, it is reasonable to conclude that the related central regions of the Gag proteins of spuma- and orthoretroviruses, as well as having conserved functions have arisen as a result of genetic divergence from a common, double domain ancestor.

### Gag assembly

The capacity to form an assembled lattice is a key feature of retroviral Gag proteins. These structures have been well characterised for mature orthoretroviruses [58], though the versions present in immature viruses remain relatively poorly defined [59–61]. Nevertheless it is clear that the formation of CA hexamers is vital for the assembly process. By contrast, there is much less information available regarding spumavirus Gag mediated assembly. It has been demonstrated that PFV-Gag-NtD self-associates into dimers [43]. Our findings now identify PFV-Gag (NtD_CEN_-CtD_CEN_) that is structurally related to orthoretroviral CA, has the functional properties of a protein involved in capsid assembly and moreover, FV polyhedral core structure is dependant on PFV-Gag (NtD_CEN_-CtD_CEN_) structural integrity.

A clue to how PFV Gag might assemble is revealed by the structure of PFV-CtD_CEN_ (**Fig 4**). In isolation PFV-CtD_CEN_ forms weak dimers, K_D_ = 0.9 μM (**Fig 3**) through homotypic interactions mediated by hydrophobic side chains located on helices α5 and α6. This is in contrast, to the orthoretroviruses where the major CA-CtD interface is formed through homotypic interactions between residues on CA-CtD α9 that would align to α7 in PFV-CtD_CEN_ and therefore appears unrelated. Nevertheless, in the context of intact PFV-Gag, formation of these CtD_CEN_-CtD_CEN_ interactions would require conformational rearrangement to expose the α5-α6 interface that would consequently release the NtD_CEN_ domains to make further homotypic interactions. However, given we have demonstrated the capacity for CtD_CEN_ self-association it is a possibility that the CtD_CEN_-CtD_CEN_ interface is utilised by FV-Gag in CA assembly. Moreover, since Gag conformational switching is a major driver in the maturation of orthoretroviruses [59–62] the notion of a conformational change in FV Gag is certainly plausible. In further support of this notion, notably the Major Homology Region (MHR) of orthoretroviral CA is a critical driver of maturation and assembly [63–65]. The MHR comprises a strand-turn-helix structure that makes intra-hexamer homotypic CA-CtD interactions in the immature CA lattice [60, 61] and maps to α5 and α6 region of PFV-CtD_CEN_ in our alignments. Therefore, although the α5 - α6 and MHR motifs are structurally unrelated their positioning suggests a conservation of assembly function in this region.

Another prominent feature of PFV-Gag is the YxxLGL motif (**Fig 1A**) (residues Y464-L469) that is conserved in all spumaretroviruses (**Fig 1B**) and is required for particle assembly [48]. In the PFV-Gag(NtD_CEN_-CtD_CEN_) structure this motif is found at the C-terminus of α9 in CtD_CEN_ (**S5 Fig)**. The aromatic side chain of Y464 packs into a hydrophobic pocket and forms part of the core of the CtD_CEN_ helical bundle. Notably, as only Y or F are observed at this position amongst FV Gags (**Fig 1B**) the conservation is likely a result of the structural requirement for a phenyl group at this position to be buried in the hydrophobic core. By contrast, the side chains in the LGL portion of the motif are exposed and abut residues from another highly conserved PGQA motif at the N-terminal of α8 in CtD_CEN_ (residues 431–434; **Fig 1B**) to form a continuous surface hydrophobic patch located ~ 180° away from the α5 - α6 interface of CtD_CEN_ (**S5 Fig**). Given the requirement for capsid assembly, one notion is that α5 - α6 homotypic interactions and further self-association through YxxLGL/PGQA surface patch when combined with PFV-Gag-NtD dimerisation, might also give rise to hexameric assemblies analogous to those formed in orthoretroviruses. However, notably the helices containing the YxxLGL/PGQA patch actually align with α10 and α11 of orthoretroviral CA that are not major drivers of orthoretroviral CA assembly suggesting there might be an alternative packing arrangement of a spumaretroviral Gag assembly.

### Capsid formation and reverse transcription

Introduction of interface mutations V375Q and L410E/M413E or YxxLGL motif mutants [48] have little effect on virus assembly or RNA encapsidation. By contrast, dramatic effects are observed on the formation of morphologically intact cores, particle DNA content and infectivity. These seemingly incompatible data might be reconciled in the following way. It is known that initial FV capsid formation occurs within the cell cytoplasm and simultaneously viral RNA is recruited by Gag via the GR-regions [54]. Subsequently, FV Env leader peptide binds Gag to facilitate membrane targeting and particle release [45]. However, it has been demonstrated that cleavage of PFV p71-Gag to generate p68-Gag is required for the initiation of reverse transcription [66]. Furthermore, it has been shown that proteolytic processing of the Gag protein of *S*. *cerevisiae* Ty1 transposable elements that assemble in the cytoplasm is also required for reverse transcription and transposition activity [67, 68]. Although we cannot rule out that in FVs Env binding to Gag might be a trigger to conformational rearrangement, we suggest that Gag cleavage to form p68, initiates the rearrangement of Gag, resulting in the appearance of the discrete capsid layer observed by cEM. The absence of viral DNA genomes in released mutant virions **(Fig 7)** implies that this Gag rearrangement and capsid shell formation is a requirement for one or more steps in reverse transcription and may be analogous to maturation in orthoretroviruses.

### Capsid structure and restriction

Members of the Trim5α family of restriction factors block infection of cells by HIV-1, as well as other lentiviruses, gammaretroviruses and the FVs [31, 69]. Orthoretrovirus restriction requires interaction of Trim5α with the CA component of Gag in the context of an assembled capsid shell [32, 70] consistent with the genetic mapping within CA of the amino-acid determinants for restriction specificity [71, 72]. It appears that CA-hexamers, the basic building block for core assembly, represent the primary target for Trim5α restriction [73, 74] and a similar picture is emerging for Fv1 [75, 76]. However, given the apparent lack of sequence identity between orthoretroviral and FV Gag proteins, it has been unclear how such restriction factors might recognise and restrict FVs. Indeed, the molecular determinants for Trim5α restriction of FVs seem to map to the N-terminal region of FV Gag [43]. Our structural analysis of PFV Gag now reveals that FVs also contain a CA region comprising two domains with folds related to the NtD_CA_ and CtD_CA_ of orthoretroviral Gag. This might suggest a similar mechanism for FV recognition by restriction factors where self-association of the central region through Gag CtD_CEN_ interactions in combination with dimerisation through the Gag N-terminal region [43] could also form hexameric arrays that are targeted by Trim5α. More detailed structural studies will be required to answer this question.

## Methods

### Protein Expression and purification

The DNA sequences coding for PFV-Gag residues 300–477, 300–381 (NtD_CEN_), and 381–477 (CtD_CEN_) were amplified by PCR from template plasmid pcziGag4 [77] containing the PFV Gag gene. PCR products were inserted into a pET22b expression vector (Novagen) using the NdeI and XhoI restriction sites in order to produce C-terminal His-tag fusions. The correct sequence of expression constructs was verified by automated DNA sequencing (GATC Biotech). His-tagged PFV constructs were expressed in the *E*. *coli* strain Rosetta 2 (DE3) and purified using Ni-NTA affinity (Qiagen) and size exclusion chromatography (SEC) on Superdex 75 (GE healthcare). For NMR studies proteins were grown in minimal media supplemented with ^15^NH_4_Cl, ^13^C-Glucose and/or ^2^H_2_O and purified as described.

### NMR Spectroscopy

All NMR experiments were carried out at 298 K on Bruker Avance 600-, 700-, 800-, and 950-MHz spectrometers. ^1^H/^2^H, ^13^C ^15^N-labeled PFV-Gag samples, PFV-Gag(300–477) (residues 300–477) and PFV-Gag CtD_CEN_ (residues 381–477) were prepared in buffer containing 20mM Tris-HCl, 20 mM NaCl, 0.5 mM TCEP pH 7.0. Protein concentrations for the NMR experiments were ~300 μM for PFV-Gag(300–477) and 1.6 – 2 mM for PFV-Gag CtD_CEN_. ^1^H, ^13^C and ^15^N resonance assignments for protein backbone were obtained from three-dimensional HNCA, HN(CO)CA, HNCACB, HN(CO)CACB, CBCA(CO)NH, HNCO, HN(CA)CO experiments. For side-chain chemical shift assignments 3D HBHA(CO)NH, CC(CO)NH, H(CCO)NH, (H)CCH-TOCSY, and CCHTOCSY spectra were also acquired. In addition, aromatic side-chain resonances were assigned from the analysis of the ^1^H-^13^C HSQC tuned to aromatic carbons, 2D (HB)CB(CGCD)HD, 2D (HB)CB(CGCDCE)HE as well as 3D ^13^C-edited NOESY-HSQC tuned to aromatic carbons. Inter-proton distance restraints for structural calculations were obtained from 3D ^13^C-edited NOESY-HSQC and ^15^N-edited NOESY-HSQC spectra recorded using a 100 ms mixing time. The dimer interface of PFV-Gag CtD_CEN_ was identified by intermolecular distance restraints using ^13^C/^15^N-filtered ^13^C-edited NOESY spectra. The 3D-filtered spectra were obtained using an asymmetrically labelled dimer of PFV-Gag CtD_CEN_ prepared by mixing equimolar unlabelled protein with uniformly ^13^C/^15^N-labeled protein (1.6 mM total protein concentration). For residual dipolar coupling (RDC) measurements, weakly aligned ^15^N-labelled samples of PFV-Gag(300–477) (200 μM) and PFV-Gag CtD_CEN_ (2 mM) were prepared by the addition of 10mg/ mL filamentous phage Pf1 (ASLA Biotech Ltd, Latvia). 1D NH RDCs were measured using the In-Phase and Anti-Phase method [78]. The RDC values were obtained by subtracting the reference value in isotropic solution. All spectral data were processed with NMRPipe [79] and analysed with CARA [80].

### Protein structure determination

The solution structures for PFV-Gag(300–477) and the PFV-Gag CtD_CEN_ dimer were calculated using the program ARIA (Ambigious Restraints for Iterative Assignment v 2.3) [81]. Nine iterations of progressive assignment and structure calculation combined with NOE distance restraints, hydrogen bonds, dihedral angle restraints, predicted by the TALOS program [82] and RDC measurements were employed in a simulated annealing protocol. For the PFV-Gag CtD_CEN_ homodimer the inter-proton NOE-derived distance restraints present in the filtered NOESY experiments were defined as intermolecular and the corresponding NOEs removed from the 3D ^13^C-NOESY-HSQC.

Initial structures were used to determine the axial and rhombic components of the alignment tensors with the program MODULE [83]. Subsequently, the RDC restraints were added in the final refinement stage of structure calculations. Only data for residues located in rigid secondary structure elements (^1^H-^15^N NOE > ~0.75) were employed. A final ensemble of the 20 lowest energy structures derived from 100 calculated structures and refined in an explicit water box in the last iteration was selected. The superimposition of the 20 lowest-energy structures and the ribbon diagram of one representative PFV-Gag(300–477) and one PFV-Gag CtD_CEN_ dimer structure are shown in **S1A** and **S4A Figs.** The quality of the calculated structure ensembles were assessed and validated with the Protein Structure Validation Suite-PSVS [84] and Procheck-NMR [85]. For the final 20 lowest-energy NMR structures, no distance or torsional angle restraint was violated by more than 0.5 Å or 5°, respectively. Structure determination details are summarised in **Table 1**.

### ^15^N Relaxation measurements

The backbone ^15^N relaxation parameters of the spin-lattice relaxation time T_1_, the spin-spin relaxation time T_2_ and the steady-state heteronuclear ^1^H-^15^N NOE relaxation were determined at 25°C on a 700 MHz spectrometer using a ^15^N-labeled NMR samples for PFV-Gag(300–477). The time delays used for T_1_ experiments were 10, 50, 100, 200, 400, 500, 750, 1000, and 1400 ms, and those for T_2_ experiments were 8, 16, 32, 48, 64, 80, 96, 112, 128 and 160 ms. The T_1_ and T_2_ relaxation data were obtained by fitting the individual peak intensities using nonlinear spectral lineshape modelling and fitted to single exponential using routines within NMRPipe [79]. ^1^H-^15^N NOE values were calculated from peak intensity ratios obtained from spectra with and without ^1^H saturation prior to the ^15^N excitation pulse.

### Structure alignment and comparisons

The protein structure comparison service (SSM) at the European Bioinformatics Institute (http://www.ebi.ac.uk/msd-srv/ssm/) was used to perform initial searches for structural homologues in the PDB. PFV-Gag NtD_CEN_ and CtD_CEN_ were superimposed upon orthoretroviral CA NtD and CtDs using SUPERPOSE [51] from the ccp4 program package. The fit qualities based on rmsd of Cα positions were ranked using the Q-score. Structural alignments were also produced using the SAP program [53] that uses a local structural environment based comparison that is less sensitive to local structural variation than the raw rmsd measure. The significance of the SAP comparisons were assessed using customized "decoy" models to provide a background of scores against which the comparison of the native domain structures could be evaluated [52]. A representative selection of five orthoretroviruses for which both NtD_CA_ and CtD_CA_ structures were available was used allowing a joint probability of their significance to be calculated for each domain pairing.

### Analytical Ultracentrifugation

Sedimentation velocity experiments were performed in a Beckman Optima Xl-I analytical ultracentrifuge using conventional aluminium double sector centrepieces and sapphire windows. Solvent density and the protein partial specific volumes were determined as described [86]. Prior to centrifugation, samples were prepared by exhaustive dialysis against the buffer blank solution, 20 mM Tris-HCl pH 8, 150 mM NaCl and 0.5 mM TCEP (Tris Buffer). Centrifugation was performed at 50,000 rpm and 293 K in an An50-Ti rotor. Interference data were acquired at time intervals of 180 s at varying sample concentration (0.5–2.0 mg/ml). Data recorded from moving boundaries was analysed in terms of the size distribution functions C(S) using the program SEDFIT [87–89].

Sedimentation equilibrium experiments were performed in a Beckman Optima XL-I analytical ultracentrifuge using aluminium double sector centrepieces in an An-50 Ti rotor. Prior to centrifugation, samples were dialyzed exhaustively against the buffer blank (Tris Buffer). After centrifugation for 30 h, interference data was collected at 2 hourly intervals until no further change in the profiles was observed. The rotor speed was then increased and the procedure repeated. Data were collected on samples of different concentrations of PFV-Gag(300–477) and PFV-Gag CtD_CEN_ at three speeds and the program SEDPHAT [90, 91] was used to determine weight-averaged molecular masses by nonlinear fitting of individual multi-speed equilibrium profiles to a single-species ideal solution model. Inspection of these data revealed that the molecular mass of PFV-Gag(300–477) showed no significant concentration dependency and so global fitting incorporating the data from multiple speeds and multiple sample concentrations was applied to extract a final weight-averaged molecular mass. For PFV-Gag CtD_CEN_ the molecular masses showed significant concentration dependency and so global fitting of a monomer-dimer equilibrium model incorporating the data from multiple speeds and multiple sample concentrations was applied to extract the dimerisation association constant (K_A_).

### Electron cryo-tomography and image analysis

PFV Wild type and the Gag central domain mutants were examined by cryo-electron tomography. In summary, 2 μL stock virus solution was mixed with 10-nm gold particles (British-Biocell) diluted in buffer PBS and the total 2.5 μL solution was applied to amylamine glow-discharged 200 mesh copper Quantifoil (R2/2) grids in the environment chamber (4°C, 100% RH) of a Vitrobot Mark III (FEI), blotted on both sides with a double layer of paper for 4 seconds before plunging into liquid ethane. The frozen grids were transferred to a Gatan 626 cryo tomography holder and inserted into the FEI Spirit TWIN microscope operated at 120keV with a tungsten filament source. Images were recorded unbinned at a nominal magnification of 30,000(7Å/pixel) on a 2Kx2K Eagle CCD camera at -2.5 μm defocus. Tilt series for tomography were recorded automatically using Serial EM from 0 to ±60° in 2° steps, typically with a total dose less than 70 e^-^/Å^2^. Tomographic tilt series were aligned using IMOD software [92]. Alignment initially used cross-correlation and then used gold particles as fiducials. Reconstructed 3D volumes were generated by back-projection as well as SIRT method. For better visualization, individual virus particles were extracted from the whole tomograms and 50Å thick sections are shown in Fig 6.

### Cells and culture conditions

The human embryonic kidney cell line 293T (ATCC CRL-1573) [93] and the human fibrosarcoma cell line HT1080 (ATCC CCL-121) [94] were cultivated in Dulbecco’s modified Eagle’s medium (DMEM) supplemented with 10% heat-inactivated fetal calf serum and antibiotics

### Recombinant plasmid DNAs

A four-component PFV vector system, consisting of the expression-optimized packaging constructs pcoPG4 (PFV Gag), pcoPE (PFV Env), pcoPP (Pol), and the enhanced green fluorescent protein (eGFP)-expressing PFV transfer vector puc2MD9, has been described previously [50, 77, 95]. In some experiments a previously described variant of the PFV Pol packaging construct with catalytically inactive reverse transcriptase (pcoPP2, Pol iRT, YVDD_312–315_GAAA mutation) was used [50]. All PFV Gag packaging constructs used in this study are based on the parental pcoPG4 vector [95]. The PFV Gag packaging constructs encoding mutant Gag protein with alterations in central domains (pcoPG4 C368A, pcoPG4 W371A, pcoPG4 V375Q, pcoPG4 W371A+V375Q, pcoPG4 L410E+M413E) were generated by recombinant PCR techniques and verified by sequencing.

### Transfection and virus production

Cell culture supernatants containing recombinant viral particles were generated by transfection of the corresponding plasmids into 293T cells using polyethyleneimine (PEI) as described previously [66, 96]. For subsequent Western blot analysis the supernatant generated by transient transfection was harvested, passed through a 0.45-μm filter and centrifuged at 4°C and 25,000 rpm for 3 h in a SW32Ti rotor (Beckman) through a 20% sucrose cushion. The particulate material was resuspended in phosphate-buffered saline (PBS). For cryo electron microscopy analysis viral particles were produced in serum-free medium and a further concentration step using Amicon Ultra 0.5 ml 100K Concentrators was included following the first concentration by ultracentrifugation through 20% sucrose similar as described recently [54].

### Infectivity analysis

Transduction efficiency of recombinant, eGFP-expressing PFV vector particles by fluorescence marker-gene transfer assay was analyzed 72 h post-transduction as described previously [54, 95, 97]. All transduction experiments were performed at least twice. In each independent experiment the values obtained with the *wt* construct pcoPG4 were arbitrarily set to 100% and values obtained with other constructs were normalized as a percentage of the *wt* values.

### Western blot analysis

Cells from a single transfected 100 mm cell culture dish were lysed in detergent-containing buffer and the lysates were subsequently centrifuged through a QIAshredder column (QIAGEN). Protein samples from cellular lysates or purified particulate material were separated by SDS-PAGE on a 10% polyacrylamide gel and analyzed by immunoblotting as described previously [98]. Polyclonal rabbit antisera specific for PFV Gag [99] or residues1 to 86 of the PFV Env leader peptide (LP), [98] as well as hybridoma supernatants specific for PFV PR-RT (clone 15E10) or PFV integrase (IN) (clone 3E11) [100] were employed. After incubation with species-matched horseradish peroxidase (HRP)-conjugated secondary antibody, the blots were developed with Immobilon Western HRP substrate. The chemiluminescence signal was digitally recorded using a LAS3000 (Fujifilm) imager and quantified using ImageGauge (Fujifilm).

### Quantitative PCR analysis

Preparation of particle and cellular samples for qPCR analysis was performed as previously described [54, 96]. Primers, Taqman probes and cycling conditions for specific quantification of PFV genome are summarized in (**S3 Table**). All sample values obtained using a StepOne Plus (Applied Biosystems) qPCR machine were referred to a standard curve consisting of 10-fold serial dilutions of respective reference plasmid (puc2MD9) containing the target sequences. All sample values included were in the linear range of the standard curves with a span from 10 to 10^9^ copies. The values for the DNA or RNA content of viral particle samples obtained by the qPCR analysis were normalized for Gag content determined by quantitative WB as indicated above and are expressed as percentage of the *wt* (generated by transfection of cells with pcoPG4, pcoPP, pcoPE and puc2MD9).

## Supporting Information

S1 Fig NMR data for PFV Gag(300–477). (**A**) Family of PFV Gag(300–477) NMR structures. The protein backbone for each of the 20 conformers in the final refinement is shown in ribbon representation. The backbone is coloured from the N- to C-terminus in blue to red and α-helices are labelled sequentially. (**B**) Backbone ^15^N relaxation parameters of PFV Gag(300–477). The spin-lattice relaxation time T_1_ (top), the spin-spin relaxation time T_2_ (middle) and the steady-state heteronuclear ^1^H-^15^N NOE (lower) for each residue is plotted against sequence position. (**C**) Selected ^13^C-^1^H strips from the 3D- ^13^C-NOESY spectrum identifying NOEs at the interdomain region of PFV Gag(300–477). Representative interdomain NOEs are labelled.
(TIF)

S2 Fig Helical connectivity and topology. (**A**) Secondary structure elements in HIV-1 CA and PFV-Gag(300–477). The position of secondary structure elements in the HIV-1 and PFV sequences are highlighted above and below the sequences respectively. Helices and strands are represented by coils and arrows; HIV-1 CA-NTD and PFV Gag-NtD_CEN_ (Blue), HIV-1 CA-CTD and PFV Gag-CtD_CEN_ (red). (**B, C**) Secondary structure topology diagrams for PFV-Gag (NtD_CEN_-CtD_CEN_) (**B**) and HIV-1 CA (**C**), helices are shown are bars and strands as arrows. Secondary structure elements in PFV Gag-NtD_CEN_ and HIV-1 CA-NTD are shown in blue and PFV Gag-CtD_CEN_ and HIV-1 CA-CTD in red. The shaded box area highlights the α4-α6 inserted region in HIV-1 CA-NTD that is replaced by a connecting loop in PFV-Gag -NtD_CEN_
(TIF)

S3 Fig Concentration dependence of PFV Gag-CtD_CEN_ sedimentation. C(S) distributions derived from sedimentation velocity data recorded from PFV Gag-CtD_CEN_ at 16μM (orange), 76 μM (green) and 123 μM (blue) are shown. The proportion of fast moving 2.07 S dimer component increases with increasing concentration.
(TIF)

S4 Fig NMR data for PFV-Gag CtD_CEN_ homodimer. (**A**) Family of PFV-Gag CtD_CEN_ homodimer NMR structures. The protein backbone for each of the 20 conformers in the final refinement is shown in ribbon representation. The backbone of one monomer is coloured from the N- to C-terminus in blue to red and α-helices are labelled sequentially. The other monomer is shown in grey (**B**) Backbone ^15^N relaxation parameters of PFV Gag CtD_CEN_. The spin-lattice relaxation time T_1_ (top), the spin-spin relaxation time T_2_ (middle) and the steady-state heteronuclear ^1^H-^15^N NOE (lower) for each residue is plotted against sequence position. (**C**) Region of the PFV-Gag CtD_CEN_ 3D ^13^C-edited, ^13^C/^15^N-filtered NOESY spectrum. The intermolecular NOE correlations in the filtered spectrum involving residues at the dimer interface are indicated.
(TIF)

S5 Fig Conserved PGQA and YxxLGL motifs. **(A**) Primary sequence of PFV-Gag CtD_CEN_. The highly conserved PGQA and YxxLGL motifs are highlighted in blue and green respectively and residues at the homodimer interface (helices α5 and α6) are highlighted in red. (**B**) PFV-Gag CTD_CEN_ monomer structure. The monomer is shown in surface representation with secondary structure depicted as a ribbon. Helices α5 - α6 that form the homodimer interface in the structure are shown in red. The PGQA and YxxLGL conserved motifs that combine to form the hydrophobic patch are coloured in blue and green respectively.
(TIF)

S1 Table SSM superpose scores for structural alignments (PDF)

S2 Table Quantitation of viral cores (PDF)

S3 Table qPCR primer/probe set (PDF)
