# Supplementary figures and images for: Structure of a Spumaretrovirus Gag Central Domain Reveals an Ancient Retroviral Capsid
Source: PLoS Pathog. 2016 Nov 9;12(11):e1005981. doi: 10.1371/journal.ppat.1005981 (PMC5102385; doi:10.1371/journal.ppat.1005981)

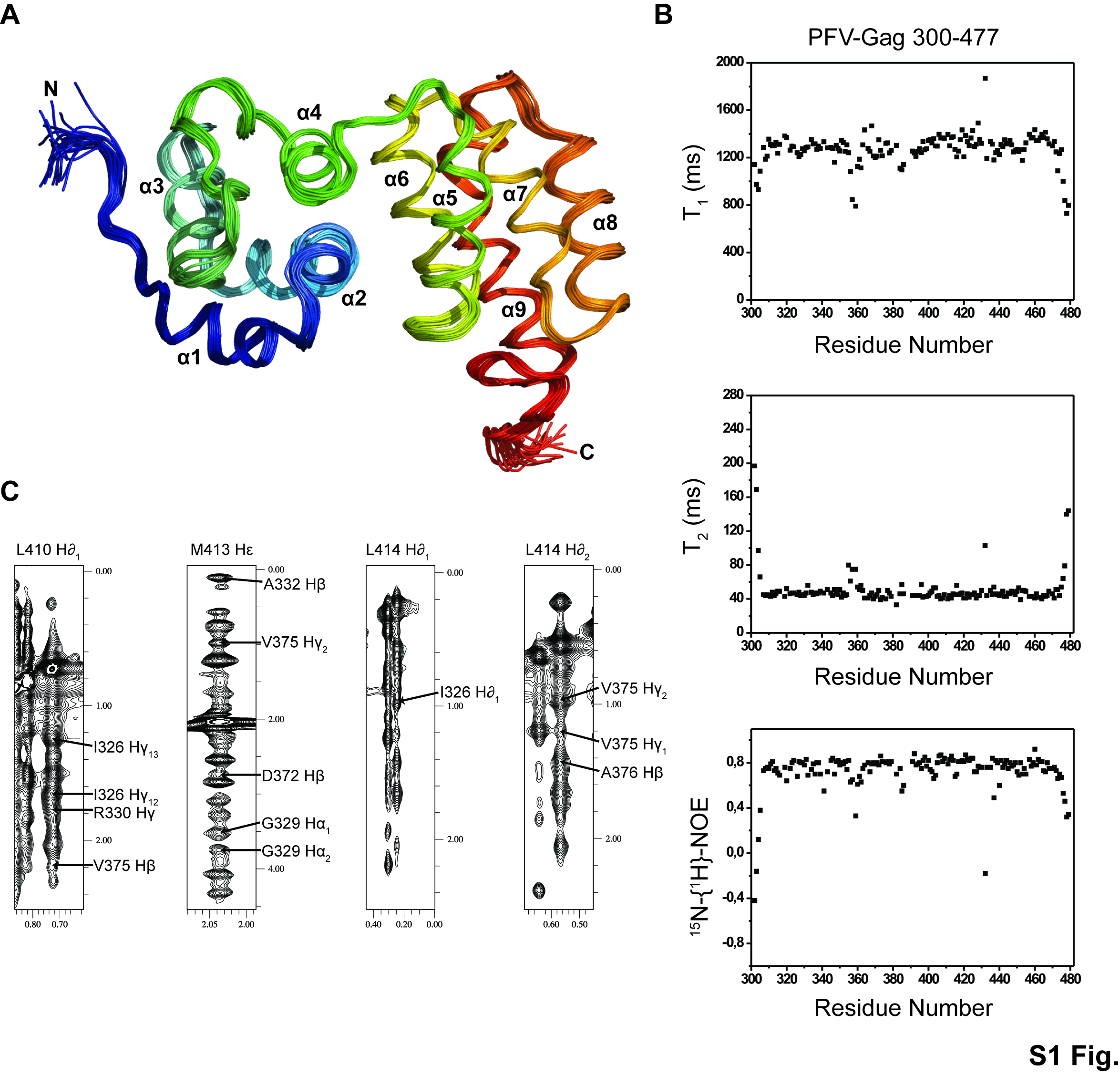

Supplement: S1 Fig — (A) Family of PFV Gag(300–477) NMR structures. The protein backbone for each of the 20 conformers in the final refinement is shown in ribbon representation. The backbone is coloured from the N- to C-terminus in blue to red and α-helices are labelled sequentially. (B) Backbone 15N relaxation parameters of PFV Gag(300–477). The spin-lattice relaxation time T1 (top), the spin-spin relaxation time T2 (middle) and the steady-state heteronuclear 1H-15N NOE (lower) for each residue is plotted against sequence position. (C) Selected 13C-1H strips from the 3D- 13C-NOESY spectrum identifying NOEs at the interdomain region of PFV Gag(300–477). Representative interdomain NOEs are labelled. (TIF) [file ppat.1005981.s001.tif]

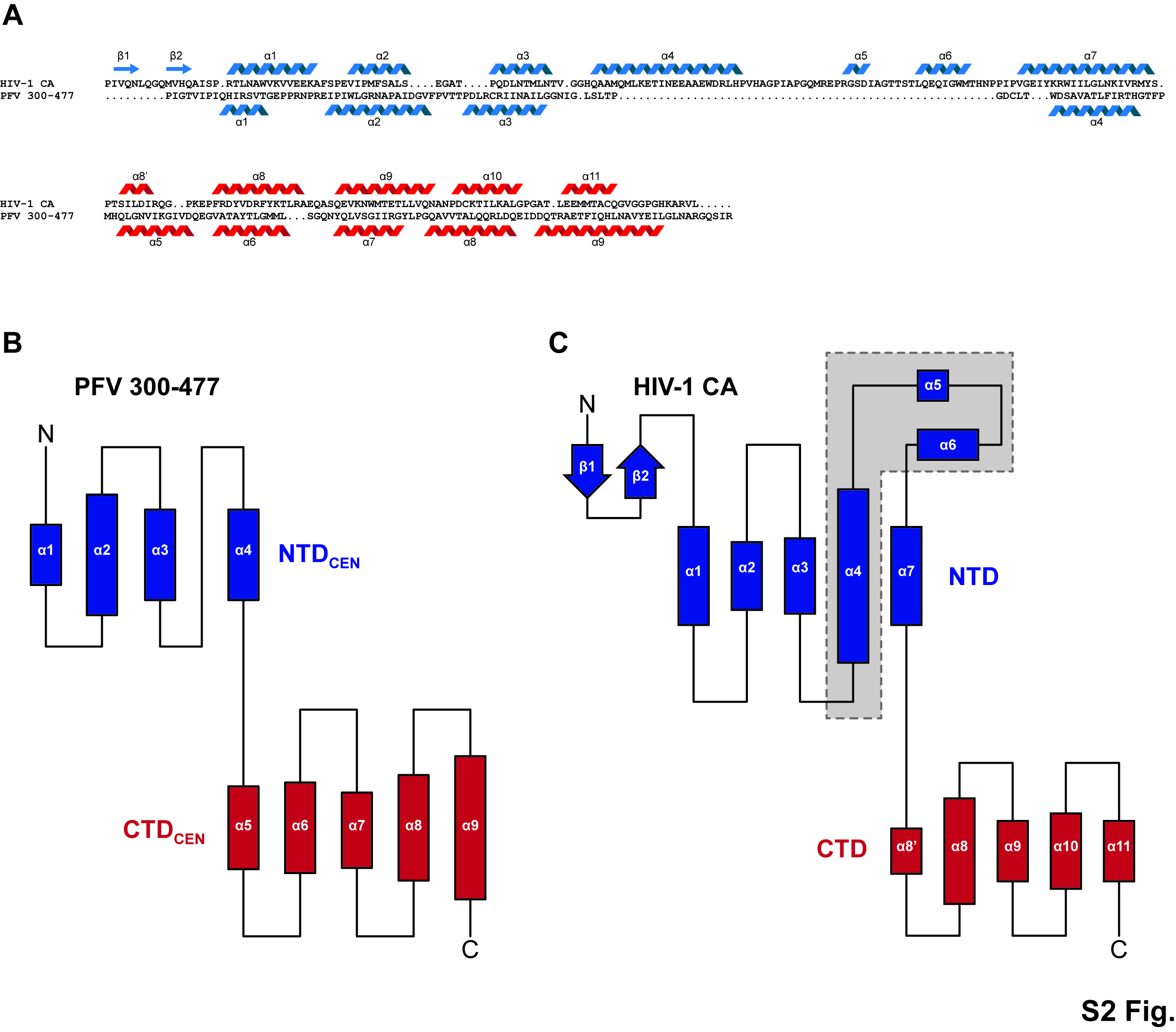

Supplement: S2 Fig — (A) Secondary structure elements in HIV-1 CA and PFV-Gag(300–477). The position of secondary structure elements in the HIV-1 and PFV sequences are highlighted above and below the sequences respectively. Helices and strands are represented by coils and arrows; HIV-1 CA-NTD and PFV Gag-NtDCEN (Blue), HIV-1 CA-CTD and PFV Gag-CtDCEN (red). (B, C) Secondary structure topology diagrams for PFV-Gag (NtDCEN-CtDCEN) (B) and HIV-1 CA (C), helices are shown are bars and strands as arrows. Secondary structure elements in PFV Gag-NtDCEN and HIV-1 CA-NTD are shown in blue and PFV Gag-CtDCEN and HIV-1 CA-CTD in red. The shaded box area highlights the α4-α6 inserted region in HIV-1 CA-NTD that is replaced by a connecting loop in PFV-Gag -NtDCEN (TIF) [file ppat.1005981.s002.tif]

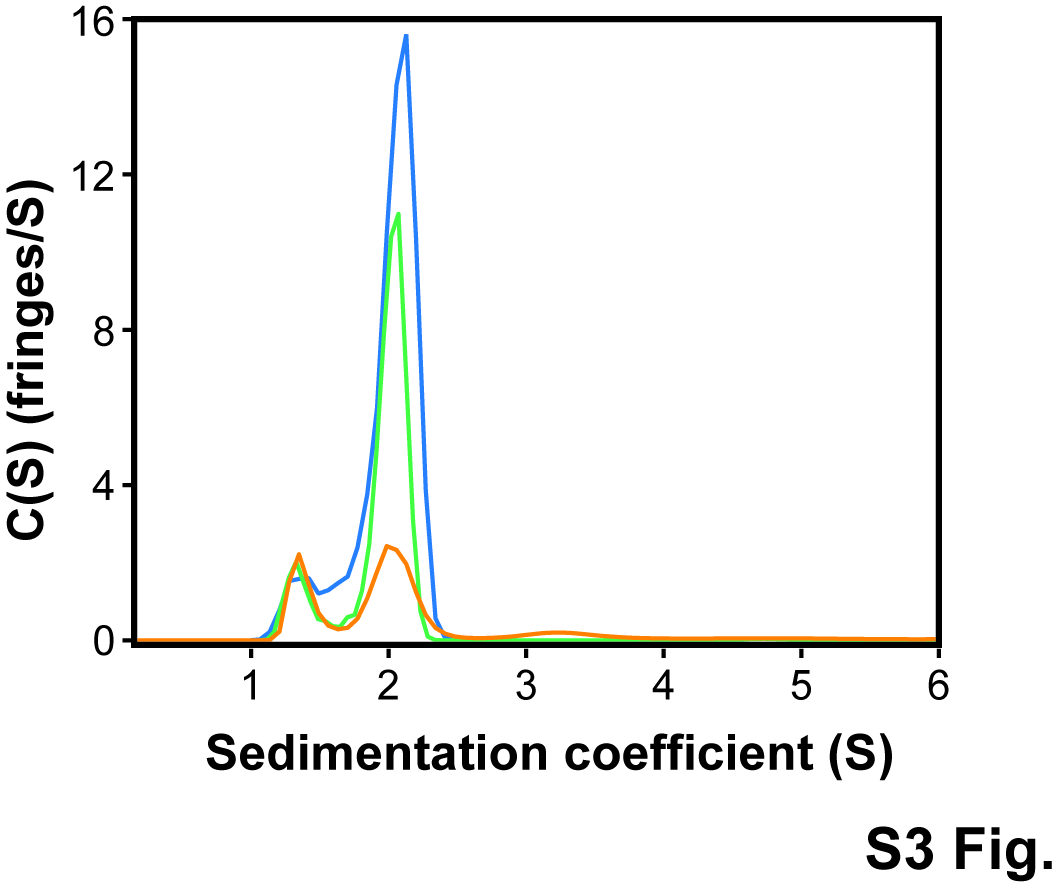

Supplement: S3 Fig — C(S) distributions derived from sedimentation velocity data recorded from PFV Gag-CtDCEN at 16μM (orange), 76 μM (green) and 123 μM (blue) are shown. The proportion of fast moving 2.07 S dimer component increases with increasing concentration. (TIF) [file ppat.1005981.s003.tif]

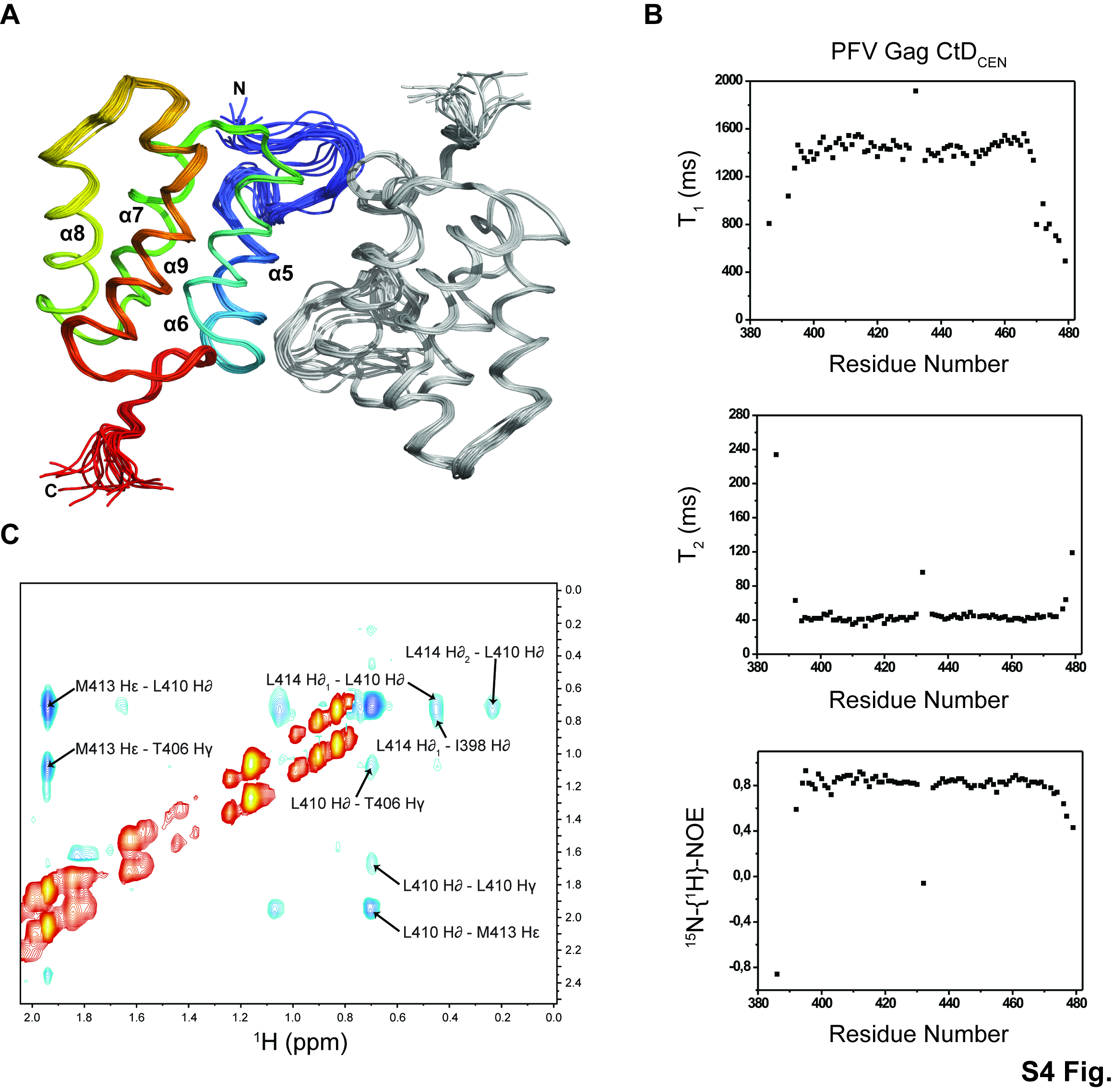

Supplement: S4 Fig — (A) Family of PFV-Gag CtDCEN homodimer NMR structures. The protein backbone for each of the 20 conformers in the final refinement is shown in ribbon representation. The backbone of one monomer is coloured from the N- to C-terminus in blue to red and α-helices are labelled sequentially. The other monomer is shown in grey (B) Backbone 15N relaxation parameters of PFV Gag CtDCEN. The spin-lattice relaxation time T1 (top), the spin-spin relaxation time T2 (middle) and the steady-state heteronuclear 1H-15N NOE (lower) for each residue is plotted against sequence position. (C) Region of the PFV-Gag CtDCEN 3D 13C-edited, 13C/15N-filtered NOESY spectrum. The intermolecular NOE correlations in the filtered spectrum involving residues at the dimer interface are indicated. (TIF) [file ppat.1005981.s004.tif]

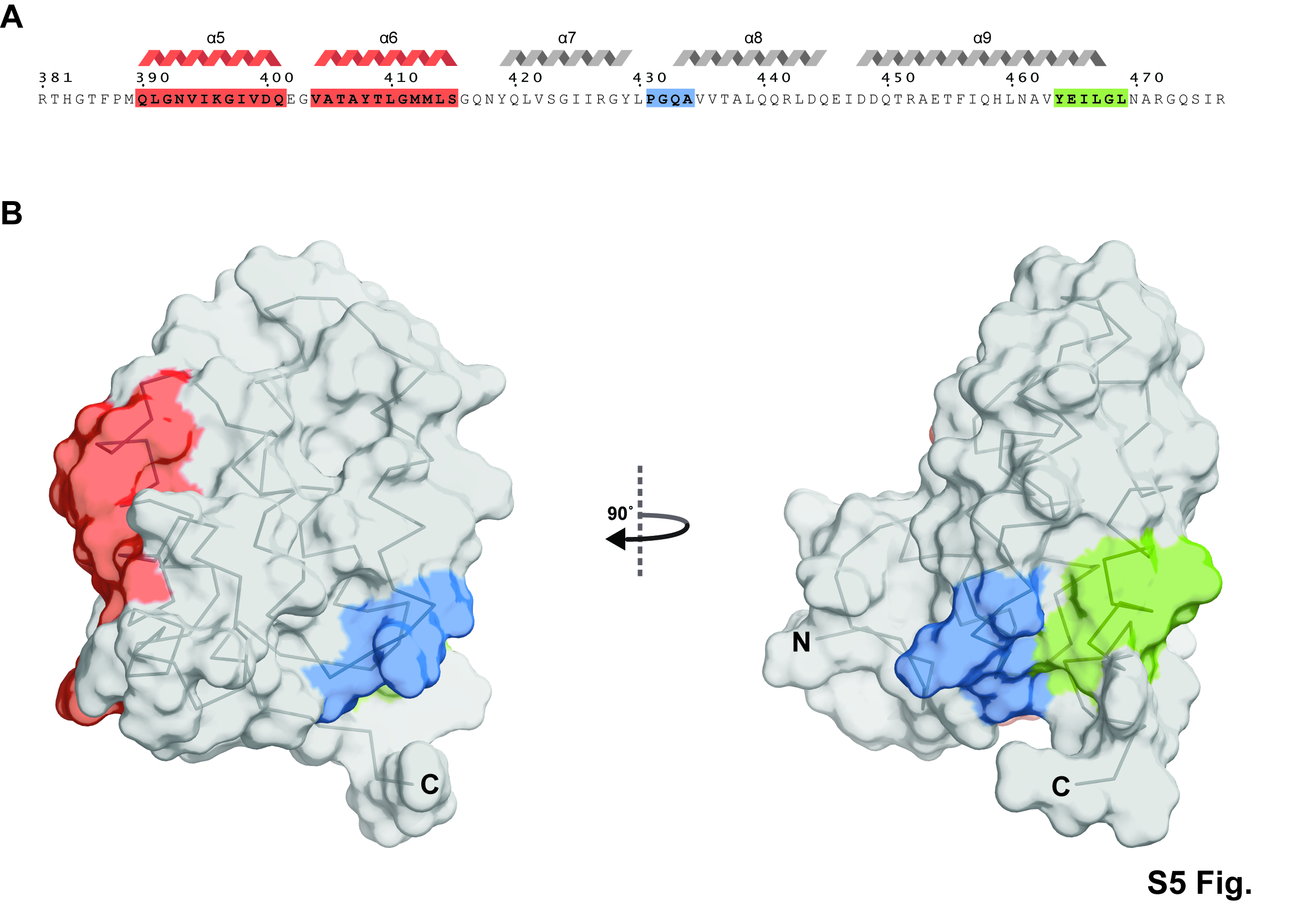

Supplement: S5 Fig — (A) Primary sequence of PFV-Gag CtDCEN. The highly conserved PGQA and YxxLGL motifs are highlighted in blue and green respectively and residues at the homodimer interface (helices α5 and α6) are highlighted in red. (B) PFV-Gag CTDCEN monomer structure. The monomer is shown in surface representation with secondary structure depicted as a ribbon. Helices α5 - α6 that form the homodimer interface in the structure are shown in red. The PGQA and YxxLGL conserved motifs that combine to form the hydrophobic patch are coloured in blue and green respectively. (TIF) [file ppat.1005981.s005.tif]
