# Supplementary material for: Structure of a Spumaretrovirus Gag Central Domain Reveals an Ancient Retroviral Capsid
Source: PLoS Pathog. 2016 Nov 9;12(11):e1005981. doi: 10.1371/journal.ppat.1005981 (PMC5102385; doi:10.1371/journal.ppat.1005981)
Supplement: S1 Table — (PDF) [file ppat.1005981.s006.pdf]

S1 Table Structural alignment scores (SSM[a])

Query	Target	Q-Score	N-align	RMSD (Å)
$PFV\text{-}NtD_{CEN}$	RSV-CtD	0.304	61	2.83
	HIV-CtD	0.252	58	3.18
	MLV-NtD	0.168	65	3.38
	RSV-NtD	0.125	58	3.26
	HIV-NtD	0.092	57	3.14
$PFV\text{-}CtD_{CEN}$	MLV-NtD	0.264	76	2.69
	RSV-NtD	0.198	72	2.90
	HIV-NtD	0.173	67	2.57
	RSV-CtD	0.113	58	3.07
	HIV-CtD	0.066	52	3.30

[a]E. Krissinel and K. Henrick (2004). Secondary-structure matching (SSM), a new tool for fast protein structure alignment in three dimensions. Acta Cryst. D60, 2256-2268.
