# Supplementary material for: Structure of a Spumaretrovirus Gag Central Domain Reveals an Ancient Retroviral Capsid
Source: PLoS Pathog. 2016 Nov 9;12(11):e1005981. doi: 10.1371/journal.ppat.1005981 (PMC5102385; doi:10.1371/journal.ppat.1005981)
Supplement: S2 Table — (PDF) [file ppat.1005981.s007.pdf]

S2 Table Quantitation of viral cores

Sample	[a]Particles	Whole core	Partial core
WT	60	35	7
V375Q	28	0	0
L410E/M413E	36	0	2
C368A	62	0	4
W371A	18	0	1

[a]Total number of tomograms recorded for WT and each mutant
