# Supplementary material for: Structure of a Spumaretrovirus Gag Central Domain Reveals an Ancient Retroviral Capsid
Source: PLoS Pathog. 2016 Nov 9;12(11):e1005981. doi: 10.1371/journal.ppat.1005981 (PMC5102385; doi:10.1371/journal.ppat.1005981)
Supplement: S3 Table — (PDF) [file ppat.1005981.s008.pdf]

S3 Table. qPCR primer/probe set

Target	Primer/ Probe	5'-3' Sequence[a]	Cycle Conditions
PFV genome (LTR R-U5)	fwd	TAAGGGTGATTGCAATGCTT	95 °C, 8 min, 1x
	rev	ATGTCTCCCTTAGCAAGGCT	95 °C, 30 s, 40x
	probe	[a]FAM-TCAATAAACCGACTTGATTCGAGAACC-BHQ1	59 °C, 30 s, 40x
			72 °C, 30 s, 40x

[a]FAM: 6-carboxyfluorescein; BHQ1: Black Hole Quencher 1
